# Medical Image Watermarking Technique for Accurate Tamper Detection in ROI and Exact Recovery of ROI

**DOI:** 10.1155/2014/984646

**Published:** 2014-09-24

**Authors:** R. Eswaraiah, E. Sreenivasa Reddy

**Affiliations:** Department of Computer Science and Engineering, Acharya Nagarjuna University, Guntur 522510, Andhra Pradesh, India

## Abstract

In telemedicine while transferring medical images tampers may be introduced. Before making any diagnostic decisions, the integrity of region of interest (ROI) of the received medical image must be verified to avoid misdiagnosis. In this paper, we propose a novel fragile block based medical image watermarking technique to avoid embedding distortion inside ROI, verify integrity of ROI, detect accurately the tampered blocks inside ROI, and recover the original ROI with zero loss. In this proposed method, the medical image is segmented into three sets of pixels: ROI pixels, region of noninterest (RONI) pixels, and border pixels. Then, authentication data and information of ROI are embedded in border pixels. Recovery data of ROI is embedded into RONI. Results of experiments conducted on a number of medical images reveal that the proposed method produces high quality watermarked medical images, identifies the presence of tampers inside ROI with 100% accuracy, and recovers the original ROI without any loss.

## 1. Introduction

Telemedicine eliminates distance hurdle and provides access to medical services available at far-away locations. It allows transmission of medical data from one location to another and enables handy and faithful interactions between patients and medical staff. This exchange of medical images imposes an important prerequisite that the medical images were not modified by unauthorized users. This prerequisite is called maintaining integrity of medical images. Conversely transmission of medical image and patient data independently through commercial networks leads to more cost and transmission time [1]. Watermarking is used to deal with the above two concerns.

Based on the medium used for hiding data inside an image, watermarking techniques are classified into two categories, namely, spatial domain and frequency domain. In spatial domain watermarking techniques [2–5], data is embedded directly into host image. In frequency domain techniques [6–8], data is embedded into transformed host image.

Another classification of watermarking techniques is reversible techniques and irreversible techniques. The original image can be obtained without loss from watermarked image with reversible watermarking techniques [7–10], while lossless recovery of original image is not possible with irreversible watermarking techniques [6]. Reversible watermarking is more suitable for medical images [11].

Based on application, watermarking techniques are categorized as robust, fragile, and hybrid. Robust watermarking techniques [6–8, 12] are used in applications where protection of copyright information of images is required, as robust watermarks sustain intentional or unintentional attacks on images. Fragile watermarking techniques [2–5, 13] are used in applications which require detection of tampers caused by unauthorized persons during transmission of images and also authorization of source of image. Hybrid watermarking techniques [14–16] are used in applications that require both privacy control and integrity control of images. These are the amalgamation of fragile and robust watermarking techniques. Here, robust watermarks are used for privacy control and fragile watermarks are used for the integrity control of image.

Most of the medical images contain two parts called ROI and RONI. From diagnosis point of view ROI part is more important. Care should be taken while hiding data into ROI part so that visual quality will not be degraded. At the same time any tampering with ROI must be identified and the original ROI must be recovered in order to avoid misdiagnosis and retransmission of medical image. The recovery data of ROI is generally embedded into RONI [3–5, 8, 16–18]. When any tamper is detected inside ROI of received watermarked medical image the tampered area of ROI is replaced with the recovery data embedded inside RONI.

In this paper, we propose a novel block based fragile medical image watermarking technique to achieve the following objectives.

1. Identifying tampered blocks inside ROI accurately using both average and variance values of blocks.
2. Recovering original ROI with zero loss, when it is tampered.
3. Detecting tampers inside ROI and recovering original ROI with simple mathematical calculations.
4. Avoiding the process of checking ROI of watermarked medical image for the presence of tampers when the ROI is not tampered.
5. Avoiding distortion in ROI of watermarked medical image by not embedding any data inside ROI.

The rest of the paper is organized into four sections.  Section 2 covers review literature, the proposed method is explained in Section 3, results are illustrated in Section 4, and finally conclusion is in Section 5.

## 2. Literature Review

So far many block based watermarking techniques were developed for identifying tampered areas inside ROI of medical images and recovering original ROI when any tamper is detected inside it. Zain and Fauzi [2] proposed a scheme, where the medical image is segmented into 8 × 8 blocks and then a mapping is established between the blocks for embedding the recovery information of each block into its corresponding mapped block. Later, each block is further divided into four subblocks of size 4 × 4 and then a 9-bit watermark is generated for each subblock. The generated 9-bit watermark of each subblock is embedded into LSBs of the first 9 pixels of the subblock in the corresponding mapped block. At receiver's end, the watermarked medical image is divided into blocks of 8 × 8 size and then the mapping between the blocks is calculated as done in embedding procedure. Later, each block is further divided into four subblocks of 4 × 4 size and then a 2-level detection scheme is applied for detecting tampered blocks. This 2-level detection scheme identifies tampered blocks, where level-1 detection is applied to subblocks of blocks and level-2 detection is applied to blocks. When a tampered block is detected, the corresponding mapped block is identified and then recovery data embedded in mapped block is extracted. This recovery data is used to replace the pixels in tampered block. Major drawbacks of this method are as follows. (1) If both block A and its mapped block B are tampered then it will not be possible to recover original image. (2) This method does not use any authentication data for the entire medical image to check directly whether the image is tampered. So, all blocks in the image have to be checked one after another to detect the presence of tampers. This checking process leads to wastage of time when the image is not tampered. (3) A tampered block cannot be recovered with original pixels of the block as this method uses average of pixels inside the block for recovering the pixels in the tampered block.

Wu et al. [6] developed two block based methods. In the second method, JPEG bit-string of the selected ROI is generated and then divided into fixed length segments. Later, the medical image is divided into blocks and then hash bits are calculated for each block excluding the block with ROI. These hash bits are used as authentication data of the blocks. In each block of image, hash bits of the block and one segment of JPEG bit-string of ROI are both embedded using robust additive watermarking technique. Then all blocks are combined to get watermarked medical image. At receiver's end, the watermarked medical image is divided into blocks as done in embedding procedure. From each block, hash bits of the block and a segment of JPEG bit-string are both extracted. For each block, hash bits are calculated and then compared with the extracted hash bits to check whether the block is tampered or not. If the block with ROI is identified as tampered then the JPEG bit-string segments extracted from all blocks are used to recover the ROI. Disadvantages of this method are as follows: (1) it is not possible to get original ROI as JPEG bit-string of ROI is used to recover ROI when it is tampered and (2) this method requires more calculations to generate recovery data of ROI and embed it into all blocks of medical image.

Chiang et al. [7] proposed two block based methods based on symmetric key cryptosystem and modified difference expansion (DE) technique. The first method has the ability to recover the whole medical image, whereas the second method has the ability to recover only ROI of medical image. In the first method, the medical image is divided into 4 × 4 size blocks and then average of each block is calculated. Later, the average values of all blocks are concatenated and then encrypted using two symmetric keys *k*1 and *k*2 in order to increase the degree of security. Then, Haar wavelet transform is applied to all blocks to identify smooth blocks. The encrypted average values of all the blocks are embedded in the identified smooth blocks. At the receiver's end, the embedded data is extracted from watermarked image and then decrypted using the keys *k*1 and *k*2 to get the average values of all blocks. Later, average values are calculated for all blocks and then compared with extracted average values to detect tampered blocks. When a tampered block is detected the pixels in tampered block are replaced with the extracted average of that block. The second method is the same as the first method except that the bits of pixels in blocks of ROI are embedded instead of average values of all blocks in the entire image. Pitfalls of these schemes are as follows: (1) the two methods require more time for embedding data into medical image as all blocks of the medical image have to be transformed into frequency domain and then smooth blocks have to be identified for embedding data and (2) the two methods are not using any authentication data for the entire ROI or the entire image to check directly whether the ROI or the entire image is tampered. So, all blocks in the ROI or in the entire image have to be checked one after another to detect the presence of tampers. This checking process leads to wastage of time when the image is not tampered.

Liew et al. [3, 4] developed two reversible block based methods. In the first method, the medical image is segmented into two regions: ROI and RONI. Later, ROI and RONI are divided into nonoverlapping blocks of sizes 8 × 8 and 6 × 6, respectively. Then, a mapping is formed between blocks of ROI to embed recovery information of each block into its mapped block. Each block in ROI is mapped to a block in RONI. This mapping is used to embed LSBs of pixels in a ROI block into its mapped RONI block. Then, the method implemented by Zain and Fauzi [2] is applied only to ROI part of the medical image for detecting tampers inside ROI and recovering original ROI. The LSBs of pixels inside ROI are replaced with its original bits that were stored inside RONI to make the scheme reversible. The second method is the same as the first method except that the removed LSBs of pixels in blocks of ROI are compressed using Run Length Encoding technique before embedding into RONI blocks. Drawbacks of the two methods are as follows. (1) If both block A and its mapped block B inside ROI are tampered then it will not be possible to recover original ROI. (2) The two methods do not use any authentication data for the entire ROI to check directly whether the ROI is tampered. So, all blocks in the ROI have to be checked one after another to detect the presence of tampers. This checking process leads to wastage of time when the ROI is not tampered. (3) A tampered block cannot be recovered with original pixels of the block as these methods are using average of pixels inside the block for recovering the pixels in the tampered block.

Memon et al. [15] implemented a hybrid watermarking method. In this method, the medical image is segmented into ROI and RONI. Then, a fragile watermark is embedded into LSBs of ROI. RONI is divided into blocks of size *N* × *N* and then a location map indicating embeddable blocks is generated. A robust watermark is embedded into embeddable blocks of RONI using integer wavelet transform (IWT). Later, the location map is embedded into LL_3_ of each block using LSB substitution method. Finally, ROI and RONI are combined to get watermarked image. At receiver's end, the watermarked medical image is segmented into ROI and RONI. Then, the robust watermark is extracted from RONI and is used for checking authentication of image. Fragile watermark is extracted from ROI and checked visually to know the presence of tampers inside ROI. Two disadvantages of this method are as follows: (1) there is no specification of how the original ROI is recovered when the ROI is tampered and (2) the time complexity of this method is more as it has to generate location map before embedding data.

Tjokorda Agung and Permana [5] developed a reversible method for medical images whose ROI size is more compared to size of RONI. In this method, the original LSBs of all pixels in medical image are collected and then LSB in each pixel is set to zero. Later, the medical image is segmented into ROI and RONI regions. Then, ROI and RONI are divided into blocks of sizes 6 × 6 and 6 × 1, respectively. A mapping is formed between blocks of ROI for storing recovery information of each ROI block into its mapped ROI block. The removed original LSBs are compressed using RLE technique and then embedded into 2 LSBs of 6 × 1 blocks in RONI. At receiver's end, the watermarked medical image is segmented into ROI and RONI as done in embedding procedure. Then, the method proposed by Zain and Fauzi [2] is applied only to ROI part to detect tampers inside ROI and recover original ROI. The original LSBs that were embedded in RONI are extracted and then restored to their positions to get the original medical image. This method has the same drawbacks as the methods proposed by Liew et al. [3, 4].

Al-Qershi and Khoo [8] developed a reversible ROI-based watermarking scheme. At sender's end, the medical image is segmented into ROI and RONI. Later, data of patient and hash value of ROI are both embedded into ROI using the technique developed by Gou et al. Compressed form of ROI, average values of blocks inside ROI, embedding map of ROI, embedding map of RONI, and LSBs of pixels in a secret area of RONI are embedded into RONI using the technique of Tian. Finally, information of ROI is embedded into LSBs of pixels in a secret area. At receiver's end, ROI information is extracted from a secret area and is used to identify ROI and RONI regions. From the identified RONI region compressed form of ROI, average values of blocks inside ROI, embedding map of ROI, embedding map of RONI, and LSB of pixels in secret area are extracted. Using the extracted location map of ROI, patient's data and hash value of ROI are extracted from ROI. Then, hash value of ROI is calculated and compared with extracted hash value. If there is a mismatch between the two hash values then the ROI is divided into 16 × 16 blocks. For each block, the average value is calculated and compared with the corresponding average value in the extracted average values. If they are not equal then the block is marked as tampered and replaced by the corresponding block of the compressed form of ROI. Two disadvantages of this method are (1) extracting the embedded data from RONI without knowing the embedding map of RONI and (2) use of compressed form of ROI as recovery data for the ROI.

Al-Qershi and Khoo [17] proposed a scheme based on two-dimensional difference expansion (2D-DE). At sender's end, the medical image is divided into three regions: ROI pixels, RONI pixels, and border pixels. Later, the concatenation of patient's data, hash value of ROI, bits of pixels inside ROI, and LSBs of border pixels are compressed using Huffman coding and then embedded into RONI using 2D-DE technique. This embedding generates a location map which will be concatenated with information of ROI and then embedded into LSBs of border pixels. At receiver's end, from border pixels in the watermarked medical image both information of ROI and location map are extracted. Using this ROI information, ROI and RONI are identified. The extracted location map is used to extract patient's data, hash value of ROI, bits of pixels inside ROI, and LSBs of border pixels from RONI. The process for detecting tampered blocks is the same as the one used in [8]. Each tampered block is replaced by the corresponding block of pixels in the extracted ROI. The LSBs of border pixels are replaced using the extracted LSBs from RONI. A major drawback of this scheme is that it is applicable to only medical images whose ROI size is very less (up to 12% of size of the entire image).

Al-Qershi and Khoo [16] developed a hybrid ROI-based method. At sender's end, the medical image is divided into three regions: ROI, RONI, and border pixels. Later, patient's data and hash value of ROI are embedded inside ROI using modified DE technique. The ROI location map along with compressed form of ROI and average intensities of blocks inside ROI is then embedded into RONI using DWT technique. Then, size of watermark that is inserted into RONI and ROI information is embedded inside border pixels using the same DWT technique. At receiver's end, ROI information is extracted from border pixels and is used to identify ROI and RONI regions. Compressed form of ROI, average intensities of blocks in ROI, and location map of ROI are extracted from the identified RONI region. Using the extracted location map of ROI, patient's data and hash value of ROI are extracted from ROI. The procedure for detecting tampered blocks and recovering ROI is the same as in [8]. Two disadvantages of this method are (1) use of compressed form of ROI as recovery information for the ROI and (2) applicability to only images whose size is at least 512 × 512.

Deng et al. [9] developed a region-based tampering detection and recovering method based on reversible watermarking and quadtree decomposition. In this method, original image is divided into blocks with high homogeneity using quadtree decomposition and then a recovery feature is calculated for each block using linear interpolation of pixels. The recovery features of all blocks are embedded as the first watermark using invertible integer transformation. Quadtree information as the second layer watermark is embedded using LSB replacement. In the authentication phase, the embedded watermark is extracted and the original image is recovered. The similar linear interpolation technique is utilized to get each block's feature. The tampering detection and localization can be achieved through comparing the extracted feature with the recomputed one. The extracted feature can be used to recover those tampered regions with high similarity to their original state. One drawback of this scheme is that original image cannot be recovered when it is tampered.

Kim et al. [19] developed a region-based tampering detection and restoring scheme for authentication and integrity verification of images based on image homogeneity analysis. This method divides the image into variable-sized blocks using quadtree decomposition and then chooses the average value of each block as the recovery feature. Some of the drawbacks identified with this method are as follows: (1) the original image cannot be recovered exactly when the region with recovery information is tampered and (2) computational complexity of the algorithm is high.

Tan et al. [20] proposed a dual layer reversible watermarking technique with tamper detection capability for medical images. This method embeds source information and encrypted location signal as layer 1 watermark into the medical image. CRC values of blocks in the medical image are used for detecting tampers and are embedded as the second layer watermark. This method is not specifying how the tampered blocks are recovered to get original image.

Most of the reviewed schemes are detecting the tampered blocks in the watermarked medical image based on average intensity of the blocks. These schemes fail in identifying the changes or tampers in any block if the values of pixels in that block are modified without changing the average value of the block. For example, if the values of pixels of a block in a watermarked medical image are as shown in Table 1 then the average intensity of the block will be 72. There is a possibility to achieve the same average intensity for the block as shown in Table 2 by changing the values of pixels. By comparing only the average values of original and modified blocks it is not possible to detect modifications done in the block accurately. So there is a need to develop a system that can detect the tampers accurately even when only the pixel values of the block are changed by keeping the average value the same.

## 3. Proposed Method

To achieve the above-mentioned objectives, we propose a novel medical image watermarking method. A medical image may contain several disjoint ROI areas in different shapes. Each ROI area is marked by a physician or by a clinician interactively and is represented by an enclosing polygon. The enclosing polygon is characterized by the number of vertices and their coordinates. In the present work, we consider medical images containing a single ROI, though the proposed method can be used with medical images containing multiple ROI areas. The outer three lines of pixels in the image are used as border of image.

**Figure 1:**
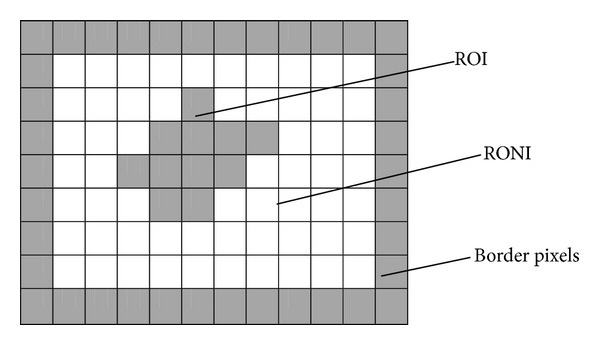
Division of medical image into three regions.

In the proposed method, the medical image is segmented into three sets of pixels, ROI pixels, RONI pixels, and border pixels as shown in Figure 1. Later, hash code of ROI is calculated using SHA-1 technique and is used to authenticate ROI. Even a change in single bit of ROI is identified using this hash code, as SHA-1 generates a unique code of size 160 bits for any input. ROI and RONI of medical image are divided into nonoverlapping blocks of sizes 4 × 4 and 8 × 8, respectively. Then, each block in ROI is mapped to a block in RONI using (1). This mapping is based on the assumption that the number of blocks in ROI is less than the number of blocks in RONI and is used to embed recovery information of each ROI block into its corresponding mapped RONI block. For each ROI block, recovery data is generated by collecting the bits of pixels inside the block. The recovery data of each ROI block is embedded into LSBs of pixels inside the corresponding mapped RONI block. Consider

(1) $$\begin{array}{c} B_{\text{RONI}}=\left[\left(k\times B_{\text{ROI}}\right)\mathrm{mod}N_{b}\right]+1, \end{array}$$

where *B*_RONI_ is block number in RONI, *k* is a secret key and is a prime number between 1 and *N*_*b*_, *B*_ROI_ is block number in ROI, and *N*_*b*_ is the number of blocks in ROI.

**Figure 2:**
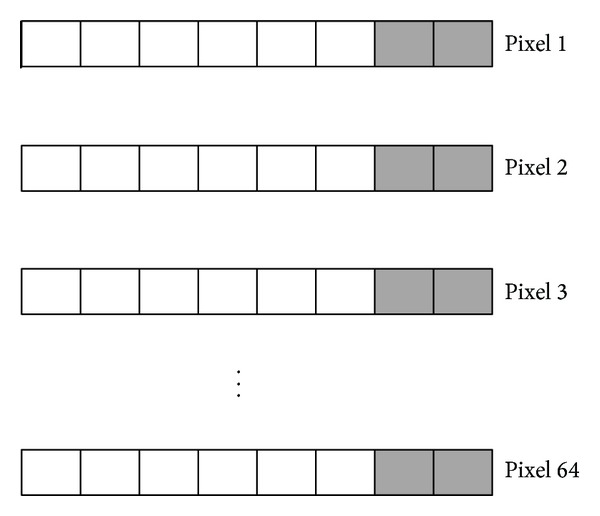
Embedding the recovery data of a ROI block into 2 LSBs of pixels in mapped RONI block.

For each 4 × 4 block of ROI in 8-bit medical images, the size of recovery data is 128 bits (collection of bits of pixels inside the ROI block). Two LSBs of each pixel in mapped RONI block are used to embed this recovery data as shown in Figure 2. Similarly, in 12-bit and 16-bit medical images the sizes of recovery data are 192 and 256 bits, respectively. Three and four LSBs of pixels in mapped RONI block are used to embed recovery data of each ROI block in 12-bit and 16-bit medical images. Finally, the information of ROI and the hash value of ROI are embedded into LSBs of border pixels, where information is defined as the number of vertices and coordinates of vertices of an enclosing polygon and border is defined as the outer three lines of pixels in the image. The detailed embedding algorithm is explained as follows.

### 3.1. Embedding Algorithm

1. Segment the medical image into three sets of pixels called ROI pixels, RONI pixels, and border pixels.
2. Calculate hash value of ROI (h1) using SHA-1 technique.
3. Divide ROI into nonoverlapping blocks of size 4 × 4 pixels.
4. Divide RONI into nonoverlapping blocks of size 8 × 8 pixels.
5. Map each ROI block to a block in RONI using (1), assuming that the number of blocks in ROI is less than the number of blocks in RONI.
6. Collect bits of 16 pixels inside each ROI block as recovery data.
7. Embed recovery data of each ROI block into 2 or 3 or 4 LSBs of pixels in mapped RONI block, depending on bit depth.
8. Encrypt the collection of bits indicating hash value (h1) and information of ROI by a secret key k1.
9. Embed the encrypted bits into the LSBs of border pixels.

The watermarked medical image is now ready to send through network to other medical practitioners at remote locations.

At receiver's end, both information and hash value of ROI are extracted from LSBs of border pixels of the received watermarked medical image. With the extracted ROI information, pixels of ROI and RONI are identified in the watermarked medical image. Then, hash value of ROI is calculated and compared with extracted hash value in order to detect the presence of tampers inside ROI of received medical image. If there is a match between the two hash values then the received medical image is authentic and is directly used for making diagnosis decisions. Mismatch between the two hash values indicates the presence of tampers inside ROI of received watermarked image. To detect tampered areas inside ROI and recover the original ROI, ROI and RONI of received watermarked image are divided into nonoverlapping blocks of sizes 4 × 4 pixels and 8 × 8 pixels, respectively, as done in embedding procedure. For each ROI block, the mapped RONI block is identified using (1). Then, bits of pixels of each ROI block are extracted from LSBs of corresponding mapped RONI block. Both average and variance of each ROI block are calculated and compared with average and variance of pixels extracted from corresponding RONI block. When a block in ROI is detected as tampered block, the extracted bits of pixels are used to recover the original ROI block. The detailed extraction algorithm is explained as follows.

### 3.2. Extraction Algorithm

1. Extract the encrypted bits from the LSBs of border pixels.
2. Decrypt the extracted bits to obtain information of ROI and hash value of ROI (h1).
3. Identify ROI pixels and RONI pixels in the received medical image by using information of ROI.
4. Calculate hash value of ROI (h2) using SHA-1 technique.
5. Compare h1 with h2.
6. Stop the extraction procedure, if h1 = h2, otherwise.
7. Divide ROI and RONI into blocks of sizes 4 × 4 and 8 × 8, respectively.
8. Repeat steps 8 to 11 for each block (*B*) inside ROI in order to identify tampered ROI blocks.
9. Calculate average (a1) and variance (v1) values of block *B*.
10. Extract bits of pixels of ROI block *B* from 2 or 3 or 4 LSBs of pixels in mapped RONI block, depending on bit depth.
11. Calculate average (a2) and variance (v2) of extracted pixels values.
12. Mark the ROI block B as tampered if a1 ≠ a2 or v1 ≠ v2.
13. Replace each tampered ROI block *B* with the bits of pixels, extracted from corresponding mapped RONI block, to get the original ROI block.

Now the medical image is ready for making diagnosis decisions.

## 4. Experimental Results

Experiments are conducted on around hundred medical images of 8-bit, 12-bit, and 16-bit depth and of different modalities like CT scan, MRI scan, ultrasound, and so on. Out of the hundred images, 40 medical images are CT scan, 30 medical images are MRI scan, and the remaining 30 images are ultrasound. We used the metrics called peak signal-to-noise ratio (PSNR) and weighted peak signal-to-noise ratio (WPSNR) [21] for measuring the quality of generated watermarked medical images. The formula for PSNR is as follows:

(2) $$\begin{array}{c} \text{PSNR}\left(\text{dB}\right)=10*\log\left(\frac{255^{2}}{\text{MSE}}\right)\\ \text{MSE}=\overset{x}{\underset{i=1}{\sum }}\overset{y}{\underset{j=1}{\sum }}\frac{\left(\left|A_{ij}-B_{ij}\right|\right)}{x*y}, \end{array}$$

where *x* and *y* are the width and height of the image. *A*_*ij*_ is original medical image and *B*_*ij*_ is watermarked medical image.

Higher value of PSNR and WPSNR indicates less distortion in the watermarked images. A metric called mean structural similarity index (MSSIM) [22] is used to measure the similarity between the original and the watermarked medical images. The value of MSSIM is between −1 and 1. Value 1 of MSSIM designates that the original and watermarked images are similar. To know the level of degradation in the watermarked medical image, total perceptual error (TPE) [23] metric is used. Lower value of TPE designates less degradation in the watermarked image.

**Figure 3:**
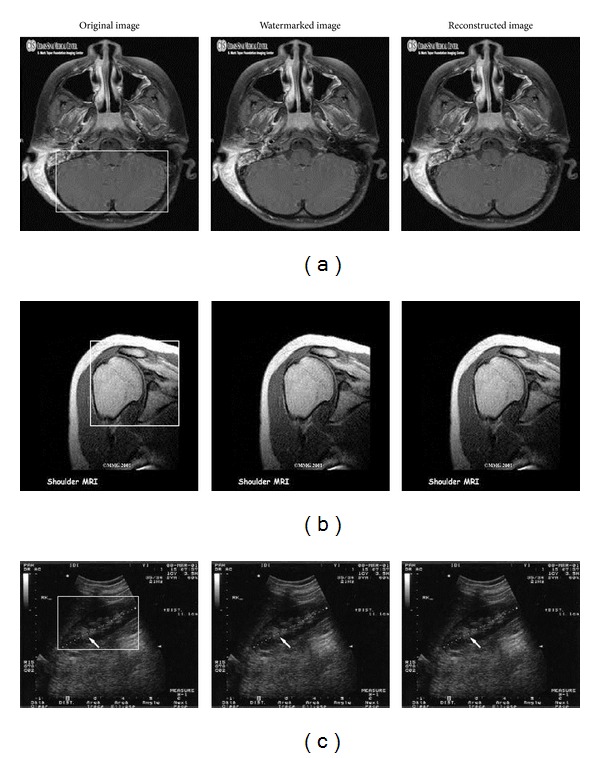
Original, watermarked, and reconstructed medical images. From top to bottom: CT scan, MRI scan, and ultrasound images.

Figure 3 shows some of the medical images used in our experiments. These images are CT scan of brain, MRI scan of shoulder, and ultrasound image of abdomen. For simulating the proposed technique, a rectangular shaped ROI is considered in each medical image. Figure 3 also shows the watermarked medical images and the reconstructed medical images. There is no considerable visual difference between original, watermarked, and reconstructed images.  Table 3 depicts the results of experiments conducted on the three medical images that are shown in Figure 3. The average results obtained by conducting experiments on the hundred medical images are shown in Table 4.

In the proposed technique, the values of PSNR and WPSNR of watermarked and reconstructed medical images are greater than 40 dB. If the PSNR and WPSNR values of the watermarked and reconstructed medical image are above 40 dB then the medical image watermarking technique is said to be effective [24]. The apparent change in the structural information of the watermarked medical images is immaterial as the MSSIM values of all medical images are very near to 1. Similarly, the low TPE values indicate less degradation in the watermarked medical images.

The intruders are prevented from obtaining hash value and information of ROI by encrypting it with a secret key before embedding inside border. Some of the state-of-the-art techniques [2–5] are not using any authentication data like hash value of ROI to check directly whether the ROI is tampered or not. So, all blocks inside ROI have to be checked one after the other to detect the presence of tampers. This checking process leads to wastage of time when the watermarked medical image is not tampered. Such wastage of time is not incurred in the proposed method as it is using hash value of ROI to directly check whether the ROI is tampered.

**Figure 4:**
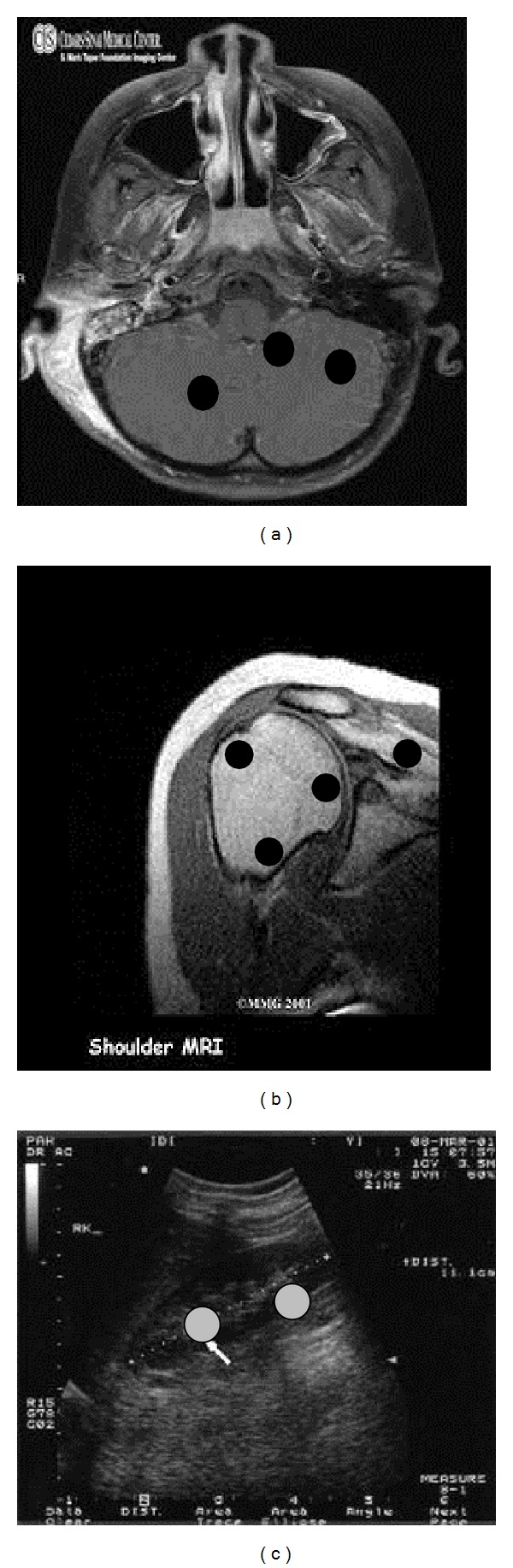
Watermarked medical images (from left to right: CT scan, MRI scan, and ultrasound) with tampers inside ROI.

**Figure 5:**
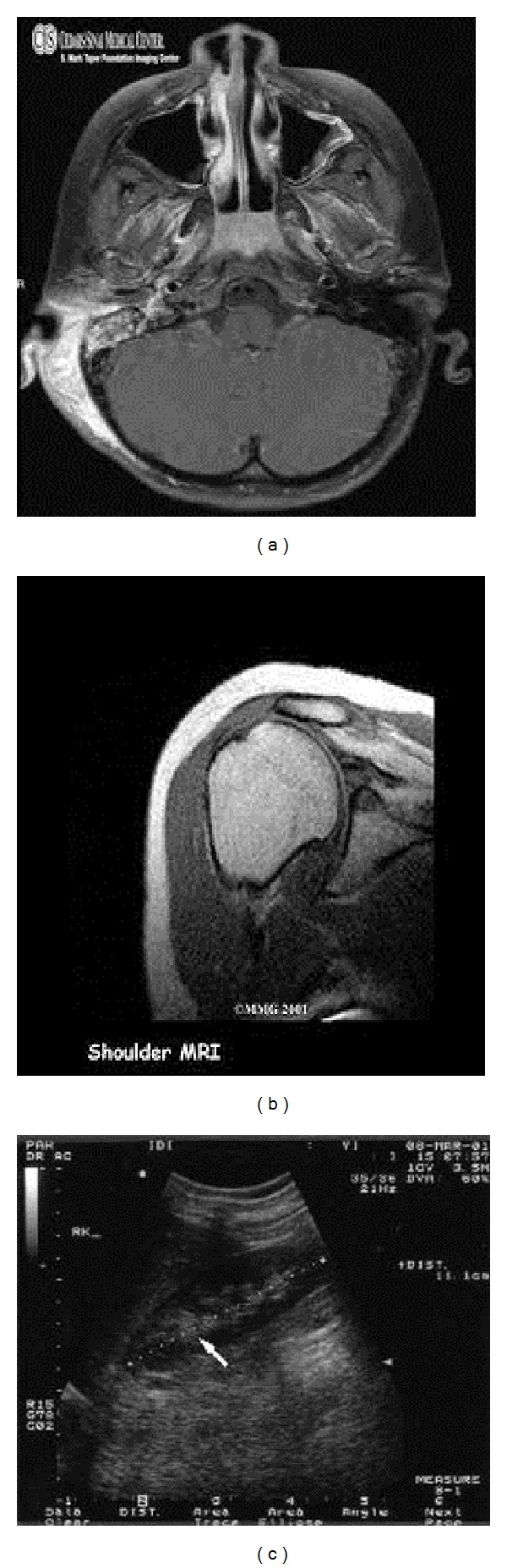
Recovered medical images (from left to right: CT scan, MRI scan, and ultrasound).

As shown in Figure 4, we induced some tampers into ROI of the watermarked medical images for testing the performance of the proposed scheme in terms of detecting tampered or modified areas inside ROI and recovering original ROI. The proposed method identified all the tampered locations inside ROI with 100% accuracy and recovered the original ROI with no loss as shown in Figure 5. In medical images, the LSBs of pixels inside RONI and border are zero. So, the LSBs of pixels in RONI and border are set to 0 after extracting the embedded data from them.  Table 5 shows comparison between the proposed scheme and the reviewed schemes.

The proposed method is developed on the assumption that the intruders generally try to modify only the significant part, ROI, in the medical images during their transmission. So, identifying changes inside ROI and recovering original ROI must be done before using the medical image for making diagnosis decisions. The proposed method can be used with medical images whose pixels are represented using 8 or 12 or 16 bits and with medical images of different modalities like CT scan, MRI scan, ultrasound, and so on. The RONI and border parts are not recovered exactly as LSBs of all pixels in RONI and border are set to bit 0 after extracting embedded data from them. This limitation does not affect the efficiency of the method as RONI and border parts of medical images are insignificant in the process of diagnosis decision making. It can only be used with medical images whose ROI size is small (up to 25% of the entire medical image). It is not robust against common attacks and image manipulation operations. This method can recover original ROI only when the RONI and border of the watermarked medical image are not attacked or modified by intruders. As intruders generally try to modify the ROI of the medical images during their transmission, this method emerges as a significant alternative in the field of medical image transmission.

## 5. Conclusion

In this paper, we proposed a block based fragile medical image watermarking technique for tamper detection and recovery. It is evident from the values of PSNR, WPSNR, MSSIM, and TPE that the proposed method produces high quality watermarked medical images. Embedding distortion inside ROI of watermarked medical images is zero as no data is embedded into ROI. The proposed method accurately identifies and localizes tampered blocks inside ROI using average and variance values of blocks. Original ROI with zero loss is recovered as the pixels in tampered blocks are replaced with original pixel values. The proposed method uses simple mathematical calculations for generating authentication and recovery data, identifying tampered blocks inside ROI, and recovering original ROI. This scheme does not check the presence of tampers inside ROI when the extracted hash value of ROI matches recalculated hash value of ROI. But some of the reviewed schemes are checking the presence of tampers without ascertaining whether or not the ROI or the entire medical image is tampered.

For future enhancement, we try to extend the method for medical images with large size ROI and to sustain common attacks and image manipulations.

## Figures and Tables

**Table 1:** A 4 × 4 block in a medical image.

66	70	84	71
71	83	72	65
79	68	74	69
80	73	80	75

**Table 2:** Modified 4 × 4 block of the medical image.

80	64	73	68
80	69	79	73
66	82	71	70
77	83	82	63

**Table 3:** Results of embedding data in three medical images of different modalities.

Modality	Size of image	Bit depth	Size of ROI	Number of blocks in ROI	PSNR	WPSNR	MSSIM	TPE
CT	336 × 406	12 bits	144 × 168	1512	53.36	54.15	0.9575	0.0549
MRI	480 × 512	16 bits	208 × 216	2808	51.52	53.44	0.9327	0.0828
US	309 × 255	8 bits	132 × 106	874	56.82	58.13	0.9854	0.0346

**Table 4:** Average performance of the proposed method.

Modality of image	Average PSNR	Average WPSNR	Average MSSIM	Average TPE
CT scan	50.26	52.81	0.9325	0.0490
MRI scan	52.13	54.65	0.9246	0.0682
Ultrasound	55.47	56.42	0.9612	0.0301

**Table 5:** Comparison between the proposed scheme and reviewed schemes.

Scheme	ROI-based	Embedding distortion inside ROI	Spotting tampers inside ROI	Accurate identification of tampered blocks	Recovery of tampered blocks inside ROI or image
Zain and Fauzi [2]	No	—	No	No	With average intensity of blocks
Wu et al. [6]	Yes	No	No	No	With JPEG compressed form of ROI
Chiang et al. [7]	Yes	No	No	No	With average intensity of blocks
Liew and Zain [3], Liew et al. [4]	Yes	Yes	No	No	With average intensity of blocks
Memon et al. [15]	Yes	Yes	No	No	No
Tjokorda Agung and Permana [5]	Yes	Yes	No	No	With average intensity of blocks
Al-Qershi and Khoo [8]	Yes	Yes	Yes	No	With compressed form of ROI
Al-Qershi and Khoo [17]	Yes	No	Yes	No	With original pixels of blocks
Al-Qershi and Khoo [16]	Yes	Yes	Yes	No	With compressed form of ROI
Deng et al. [9]	No	—	No	No	With linear interpolation of pixels of blocks
Kim et al. [19]	No	—	No	No	With average intensity of blocks
Proposed method	Yes	No	Yes	Yes	With original pixels of blocks

## References

[1] Coatrieux G, Montagner J, Huang H, Roux C Mixed reversible and RONI watermarking for medical image reliability protection.

[2] Zain JM, Fauzi ARM Medical image watermarking with tamper detection and recovery.

[3] Liew S-C, Zain JM Reversible medical image watermarking for tamper detection and recovery.

[4] Liew S-C, Liew S-W, Zain JM (2011). Reversible medical image watermarking for tamper detection and recovery with Run Length Encoding compression. *World Academy of Science, Engineering & Technology*.

[5] Tjokorda Agung BW, Permana FP Medical image watermarking with tamper detection and recovery using reversible watermarking with LSB modification and Run Length Encoding (RLE) compression.

[6] Wu JHK, Chang R-F, Chen C-J (2008). Tamper detection and recovery for medical images using near-lossless information hiding technique. *Journal of Digital Imaging*.

[7] Chiang K-H, Chang-Chien K-C, Chang R-F, Yen H-Y (2008). Tamper detection and restoring system for medical images using wavelet-based reversible data embedding. *Journal of Digital Imaging*.

[8] Al-Qershi OM, Khoo BE Authentication and data hiding using a reversible ROI-based watermarking scheme for DICOM images.

[9] Deng X, Chen Z, Zeng F, Zhang Y, Mao Y (2013). Authentication and recovery of medical diagnostic image using dual reversible digital watermarking. *Journal of Nanoscience and Nanotechnology*.

[10] Lei B, Tan EL, Chen S, Ni D, Wang T, Lei H (2014). Reversible watermarking scheme for medical image based on differential evolution. *Expert Systems with Applications*.

[11] Luo X, Cheng Q, Tan J A lossless data embedding scheme for medical images in application of e-diagnosis.

[12] Eggers JJ, Bauml R, Tzschoppe R, Girod B (2003). Scalar Costa scheme for information embedding. *IEEE Transactions on Signal Processing*.

[13] Nisar AM, Gilani SAM NROI watermarking of medical images for content authentication.

[14] Giakoumaki A, Pavlopoulos S, Koutsouris D (2006). Multiple image watermarking applied to health information management. *IEEE Transactions on Information Technology in Biomedicine*.

[15] Memon NA, Chaudhry A, Ahmad M, Keerio ZA (2011). Hybrid watermarking of medical images for ROI authentication and recovery. *International Journal of Computer Mathematics*.

[16] Al-Qershi OM, Khoo BE (2011). Authentication and data hiding using a hybrid ROI-based watermarking scheme for DICOM images. *Journal of Digital Imaging*.

[17] Al-Qershi OM, Khoo BE ROI-based tamper detection and recovery for medical images using reversible watermarking technique.

[18] Nyeem H, Boles W, Boyd C Utilizing least significant bit-planes of RONI pixels for medical image watermarking.

[19] Kim K-S, Lee M-J, Lee J-W, Oh T-W, Lee H-Y (2011). Region-based tampering detection and recovery using homogeneity analysis in quality-sensitive imaging. *Computer Vision and Image Understanding*.

[20] Tan CK, Ng JC, Xu X, Poh CL, Guan YL, Sheah K (2011). Security protection of DICOM medical images using dual-layer reversible watermarking with tamper detection capability. *Journal of Digital Imaging*.

[21] Ponomarenko N, Krivenko S, Egiazarian K, Astola J, Lukin V Weighted MSE based metrics for characterization of visual quality of image denoising methods.

[22] Wang Z, Bovik AC, Sheikh HR, Simoncelli EP (2004). Image quality assessment: from error visibility to structural similarity. *IEEE Transactions on Image Processing*.

[23] Watson AB DCT quantization matrices visually optimized for individual images.

[24] Chen K, Ramabadran TV (1994). Near-lossless compression of medical images through entropy-coded DPCM. *IEEE Transactions on Medical Imaging*.

